# Compatibility of toothpastes with probiotics for periodontal health: an *in vitro* and *ex vivo* study

**DOI:** 10.3389/fcimb.2026.1829275

**Published:** 2026-09-11

**Authors:** Mehraveh Saghi, Wannes Van Holm, Naiera Zayed, Katalina Lauwens, Nico Boon, Kristel Bernaerts, Wim Teughels

**Affiliations:** 1 KU Leuven, Department of Oral Health Sciences, Periodontology and Oral Microbiology, Leuven, Belgium; 2 Ghent University (UGent), Centre for Microbial Ecology and Technology (CMET), Gent, Belgium; 3 Menoufia University, Department of Pharmaceutical Microbiology and Immunology, Faculty of Pharmacy, Shibin El-Kom, Egypt; 4 KU Leuven, Department of Chemical Engineering, Chemical and Biochemical Reactor Engineering and Safety (CREaS), Leuven, Belgium; 5 Universiti Malaya, Department of Restorative Dentistry, Faculty of Dentistry, Kuala Lumpur, Malaysia; 6 Chulalongkorn University, Faculty of Dentistry, Department of Periodontology, Bangkok, Thailand

**Keywords:** biofilm, oral health, periodontitis, probiotic, toothpaste

## Abstract

**Introduction:**

Dental biofilms are associated with oral diseases such as periodontitis, peri-implantitis, halitosis, and tooth decay. These diseases are characterized by dysbiosis, an imbalance between the host and microbiome. Current treatments rely on the mechanical removal of the biofilm and suppressing the microbiome with broad spectrum antimicrobials. However, the antimicrobial componentseliminate bacteria nonselectively, which can also harm beneficial commensalbacteria. Pro-microbial strategies, particularly probiotics, hold promise forrebalancing the oral microbiome toward a symbiotic state rather than eradicatingit. *Limosilactobacillus reuteri* is one of the most researched strains for modulating the oral microbiome. However, uncertainties persist, particularly regarding the impact of toothpastes on probiotic survival and efficacy.

**Methods:**

In this study, *in vitro* experiments assessed the effects of selected toothpastes on *L. reuteri* viability, adhesion to hydroxyapatite discs, and biofilm incorporation and modulation. In the *ex vivo* experiment, the survival of *L. reuteri* was evaluated in saliva samples collected from ten volunteers after brushing with the selected toothpastes.

**Results:**

The results demonstrated that the antimicrobial effect of the selected toothpastes on the probiotics was concentration and strain dependent. The adhesion of probiotics to hydroxyapatite discs was inhibited by some toothpastes. However, compared with the control, the toothpastes did not influence *L. reuteri* colonization within multi-species biofilms and, additionally, did not influence the effect of the probiotics on multi-species biofilm composition. Saliva taken after brushing with a specific toothpaste did not decrease the probiotic viability.

**Conclusion:**

In conclusion, *in vitro*, colonization and biofilm modulation by probiotics are dependent on the toothpaste used. The survival of the probiotic in saliva was not affected by the toothpaste *ex vivo*.

## 1 Introduction

The oral microbiome is diverse and comprises different types of microbial ecologies. Some of these microbial communities develop on oral surfaces in structurally and functionally organized biofilms, also known as dental plaque (Filoche et al., 2010; Marsh and Zaura, 2017). When specific bacteria predominate in dental plaque, particular diseases emerge (Herrero et al., 2018). Periodontitis and tooth decay are among the most prevalent microbiological diseases worldwide and are associated with dental biofilms (Lovegrove, 2004). Although periodontitis is an inflammatory disease triggered by bacteria, it is not a condition attributable to a single pathogen (Darveau, 2010; Berezow and Darveau, 2011). Both oral diseases result from dysbiosis, a disturbance of the ecological balance that alters the composition of health associated microbial communities (Valm, 2019).

Controlling oral biofilms in quantity and composition is key to prevent oral diseases. Keeping the ecology of the biofilm in symbiosis is likely just as important as controlling the amount of biofilm. By brushing and interdental cleaning, one can mechanically reduce biofilms, but never completely remove them. Therefore, antimicrobial toothpastes and mouthwashes are often used (Slots, 2002; Gunsolley, 2006). However, bacteria in a biofilm are more protected against these antimicrobials than their planktonic counterparts since they are embedded in an extracellular polymeric slime matrix (Bowen et al., 2018; Cieplik et al., 2019; Chatzigiannidou et al., 2020). Because the inner layers of biofilms are better protected, the antimicrobial will primarily impact the outer layers of the biofilm (Mosaddad et al., 2019; Chatzigiannidou et al., 2020; Zayed et al., 2022). Additionally, antimicrobial products and antibiotics used in the prevention and adjunctive treatment of oral diseases generally lack specificity, affecting both pathogenic and beneficial commensal bacteria (Lauwens et al., 2025). It should therefore not be surprising that oral bacteria are becoming increasingly resistant to antimicrobials (Stewart and Costerton, 2001; Verspecht et al., 2019; Zayed et al., 2022). Therefore, alternative treatment or preventive options should be explored.

The use of probiotics, “live microorganisms which when administered in adequate amounts confer a health benefit on the host” (Hill et al., 2014), as a method to modulate the composition of oral biofilms has attracted interest (Allaker and Stephen, 2017). The previously classified *Lactobacillus* is one of the genera originating several probiotics, comprising *Lactobacillus acidophilus*, *Lacticaseibacillus rhamnosus*, *Lacticaseibacillus casei*, and *Limosilactobacillus reuteri*. *L. reuteri* are natural inhabitants of the human body (Frese et al., 2011; Mu et al., 2018) and can be found in the gastrointestinal tract, vagina, and breast milk (Douillard et al., 2013; Mu et al., 2018; Shah et al., 2024). In oral inflammatory diseases, the administration of *L. reuteri* DSM 17938 and ATCC PTA 5289 has been associated with reduced gum inflammation and a decrease in pathogens associated with periodontitis (Iniesta et al., 2012; Canut-Delgado et al., 2021). These results suggest the ability of these *L. reuteri* strains to modulate host inflammatory response, inhibit pathogenic bacteria, and prevent dysbiosis by maintaining host-microbiome balance in the oral environment (Gupta et al., 2020). Moreover, studies have demonstrated the potential role of biotics in dental caries prevention (Banakar et al., 2024) and periodontal therapy (Baddouri and Hannig, 2024). However, the scientific evidence for effective implementation of oral probiotics for oral health is still limited to this day. In particular, little is known about probiotic compatibility with commonly used toothpastes and the impact of these products on probiotic colonization.

In the current study, the compatibility of six commercially available toothpastes with different active ingredients and two commonly used *L. reuteri* probiotic strains was evaluated. The survival of the probiotics in different toothpaste slurries was assessed, followed by an investigation of their survival in saliva after brushing with selected toothpastes *ex vivo*. Probiotics’ adhesion to hydroxyapatite discs after exposure to toothpaste slurries was subsequently evaluated. Additionally, their combined effects were investigated using an *in vitro* oral biofilm model by examining total biofilm formation, probiotic incorporation, and changes in biofilm composition. Through these investigations, insights were sought into the potential of toothpastes as a means to enhance the efficacy of probiotics in promoting periodontal therapy and oral health, while also evaluating whether certain formulations may support probiotic effects rather than inhibiting the survival and colonization of probiotics.

## 2 Materials and methods

### 2.1 Bacterial species and cultures

*Limosilactobacillus reuteri* DSM 17938 (*L. reuteri* DSM) and *Limosilactobacillus reuteri* ATCC PTA 5289 (*L. reuteri* ATCC) (BioGaia, Lund, Sweden) were maintained on MRS agar under anaerobic conditions (37°C, 10% CO_2_, 10% H_2_, 80% N_2_). Overnight cultures were prepared in MRS broth (1 × 10^9^ CFU). Oral bacteria (**Supplementary Table 1**) were cultured on blood agar plates at 37°C, aerobically (5% CO_2_; only for *Aggregatibacter actinomycetemcomitans* and streptococci) or anaerobically.

### 2.2 Evaluated toothpastes

Six commercially available toothpaste products were included in this study to represent diverse active ingredients and reflect commonly used clinical formulations (**Table 1**). To mimic the oral situation and ease of manipulation, toothpaste slurries (20% (w/v) suspension, prepared by mixing one part toothpaste with four parts sterile PBS (1 in 5)) were used *in vitro* to simulate the dilution of toothpaste by saliva during use, while ensuring standardized preparation which facilitates standardized handling and reproducible exposure conditions across experimental assays (Pulfer et al., 2022). Phosphate-buffered saline (PBS) was used as a negative control, and 0.2% chlorhexidine (CHX) as a positive control.

**Table 1:** Toothpaste products included in the studies.

	Product name	Ingredients	Active/special ingredient
1	Colgate (Sensitive Pro-Relief Complete)	Calcium Carbonate, Aqua, Bicarbonate, Sodium Lauryl Sulfate, Sodium Monofluorophosphate (1450ppm F-), Aroma, Cellulose Gum, Sodium Bicarbonate, Tetrasodium Pyrophosphate, Titanium Dioxide, Benzyl Alcohol, Sodium Saccharin, Xanthan Gum, Limonene	Arginine 8%
2	Oral B (Pro-Expert Protection) Professional	Glycerin, Hydrate Silica, Sodium Hexametaphosphate, Aqua, Sodium Gluconate, Sodium Lauryl Sulfate, Aroma, Carrageenan, Trisodium Phosphate, Stannous Fluoride, Sodium Saccharin, PVP, Stannous Chloride, Xanthan Gum, Cocamidopropyl Betaine, Cinnamal, Sodium Fluoride, Eugenol, Sodium Hydroxide, CI74160, Sodium Benzoate	Stannous Fluoride
3	O7 (Active Toothpaste)	Glycerin, Aqua, Hydrated Silica, Silica, Betaine, PEG-32, Aroma, Cocamidopropyl Betaine, Cellulose Gum, Sodium Gluconate, Sodium Citrate, Sodium Saccharin, Sodium monofluorophosphate, Magnesium sulfate, Sodium carbonate peroxide, Sodium Fluoride, Sodium Methylparaben, Tocopheryl Acetate, Citric Acid, Sodium Phytate, Sodium Chloride, Limonene	Active Oxygen
4	Sunstar (Gum/Hydral)	Aqua, Hydrogenated Starch Hydrolysate, Silica, Propanediol, Betaine, Lauryl Glucoside, Polysorbate 20, Xanthan Gum, Sodium Citrate, Sodium Monofluorophosphate, Xylitol, Cocamidopropyl Betaine, Gluconolactone, Aroma, CI77891, Taurine, Sodium Benzoate, Citric Acid, Stevia Rebaudiana Extract, Sucralose, Sodium Chloride, Sodium Hyaluronate, PVP, Calcium	1.10% w/w sodium monofluorophosphate (1450 ppm fluoride)
5	DentAid (CHX) (Perio Aid intensive care)	Aqua, Glycerin, Hydroxyethylcellulose, PEG-40 hydrogenated Castor Oil, Chlorhexidine digluconate, Sodium saccharin, Aroma, CI42090	0.12% CHX
6	DentAid (CPC) Vitis (CPC)	Aqua, sorbitol, Hydrated silica, Glycerin, Titanium Dioxide, Sodium Gluconate, Sodium Lauryl Sulfate, Xanthan Gum, Xylitol, Sodium Fluoride, Menthone Glycerin acetal, Sodium Saccharin, Cetylpyridinium Chloride, Sodium Methylparaben, Lactic Acid, Potassium Acesulfame, Aroma	0.14% CPC

### 2.3 *In vitro* survival of probiotics in toothpaste slurries

The resilience of probiotics to various toothpastes was evaluated by assessing cell integrity using a dual dye viability assay and flow cytometry. Toothpaste slurries were diluted 1 in 10 in BHI broth (Difco, Detroit, USA), and 200 µL was added to the wells of 96-well plates. Serial dilutions (1 in 10) of each toothpaste slurry were made. 5 µL of the selected probiotics (to a final concentration of 10^8^ cells/mL) were added to each well. The plates were incubated under anaerobic conditions for 24h. Afterwards, flow cytometry analysis was performed using a FACSVerse cytometer (BD Biosciences, Belgium) with a blue 488-nm laser and a red 640-nm laser as described by Van Holm et al (Van Holm et al., 2023).

### 2.4 *Ex vivo* survival of probiotics in saliva after brushing with toothpastes

Prior to the study, approval was obtained from the Ethics Committee Research UZ/KU Leuven (B3222022000864).

Ten healthy volunteers were included in this study (4 males and 6 females), aged between 18 and 49 years. Inclusion criteria were: healthy volunteers, willing and able to provide informed consent. Exclusion criteria included: subjects with gingivitis or periodontitis, presence of orthodontic appliances, pregnancy or lactation, history of systemic diseases (e.g., diabetes), use of medications affecting the periodontium (e.g., bisphosphonates), chronic use of non-steroidal anti-inflammatory drugs, antibiotic use within the past 3 months, conditions requiring antibiotic prophylaxis, participation in other studies, and tobacco use.

Toothpastes contain a variety of active ingredients that may differ in their compatibility with probiotics. To identify which of these ingredients remaining in the saliva reduce probiotic survival, and to determine whether a waiting period is needed between toothbrushing and probiotic administration, we investigated the *ex vivo* survival of *L. reuteri* DSM and ATCC PTA in saliva after brushing with different toothpastes.

Ten healthy volunteers were instructed to brush with the selected toothpastes (**Table 1**) and a new manual toothbrush for 2 minutes in a random order. As a negative control, the same healthy volunteers brushed without any toothpaste. A minimum interval of 3 days was scheduled between brushing sessions with different toothpastes studied. During this washout period, participants brushed their teeth in their habitual way using their regular toothpaste as for the familiarization period (Paraskevas et al., 2007; Lauwens et al., 2025). Approximately 5 ml of saliva was collected in a sterile container at 5 fixed time points: before brushing with a selected toothpaste, 5 minutes, 30 minutes, 60 minutes, and 180 minutes after brushing. Samples were stored at -20 °C for 24 hours pending the laboratory experiments before analysis (Chevalier et al., 2007; Lauwens et al., 2025). After defrosting, the saliva samples were centrifuged and filter sterilized to remove the endogenous microbiota while retaining salivary and toothpaste components. To mimic the effects of residual concentrations of antimicrobial agents from toothpaste on probiotics, the probiotics were added to the filter-sterilized saliva at a final concentration of 10^8^ cells/mL, and incubated anaerobically for 2 hours. Afterwards the survival of the probiotics was assessed using flow cytometry as mentioned previously (Van Holm et al., 2023).

### 2.5 *In vitro* adhesion to saliva-covered hydroxyapatite

Hydroxyapatite discs (HAD, Himed, NY, USA) were incubated in a 24-well plate with 500µL of filter-sterilized saliva prepared according to Palmer et al (Palmer et al., 2001). The plates were incubated under anaerobic conditions for 24 hours at 37 °C at 170 rpm. After, the discs were rinsed with a toothpaste slurry for 1 min and dip rinsed in PBS. The HADs were transferred to a fresh 24-well plate, and 500 µL of the selected probiotics, diluted in filter-sterilized saliva to 10^8^ cells/mL, were then added to the HADs. The well plates were incubated for 2 hours at 37 °C while shaking at 170 rpm under anaerobic conditions. The discs were subsequently dip-rinsed in PBS and transferred to tubes with 1 ml of PBS. The attached probiotics were separated from the HAD by vortexing for 15 seconds and sonicating for 2 minutes, whereafter the supernatant was transferred to a 1.5ml Eppendorf tube. Viability qPCR (v-qPCR) was used to quantify the adhered probiotics (Lauwens et al., 2025).

### 2.6 *In vitro* effects on oral biofilms

To assess the effects of combining toothpastes and probiotics on oral biofilms, the multispecies (**Table 2**) biofilm model adapted from Slomka et al. was employed (Slomka et al., 2018). A Biostat-B Twin bioreactor was used to grow the multispecies community (**Table 2**) as described earlier by Herrero et al (Herrero et al., 2016), and biofilms were formed on HADs placed in 24-well plates. Samples from the bioreactor were diluted 1 in 10 in fresh Brain Heart Infusion-2 broth supplemented with 10 mM glycerol (BHI-2-gly) to grow biofilms on HADs in 24-well plates. They were incubated under microaerophilic conditions for 48h at 37 °C while shaking at 170 rpm to let biofilms develop. After 48 hours of microaerophilic biofilm formation, the biofilms were uniformly brushed with a microbrush (Micro Applicator Fine Pearl Purple, Demedent B.V., Cuijk, Netherlands) and rinsed with the different toothpaste slurries for 3 minutes at 250 rpm after which they are rinsed with PBS. This *in vitro* brushing model enables controlled and reproducible assessment of the effects of brushing with toothpaste on biofilm removal. Afterwards, the discs were transferred to fresh BHI-2-gly, and the two probiotic *L. reuteri* strains at 10^8^ cells/mL (from overnight cultures in MRS, quantified with flow cytometry) were added to the test group. Following a 2-hour microaerophilic incubation, the discs (both control and probiotic groups) were rinsed with PBS and transferred again to fresh BHI-2-gly to remove unattached probiotics as well as residual antiseptic compounds from the toothpaste, and then incubated under microaerophilic conditions. After another 24 hours, the biofilms were detached from the HAD using 0.05% trypsin-EDTA. To specifically quantify live cells, the detached cells were treated with propidium monoazide (PMAxx; Biotium, Hayward, CA, USA) at a final concentration of 0.1 mM. Samples were incubated for 10 min at room temperature in the dark, followed by 30 min of photoactivation using a GloPlate™ Blue LED Light Box (Biotium, Hayward, CA, USA). DNA was extracted using a QIAamp DNA Mini-kit (Qiagen, Hilden, Germany) according to the manufacturer’s instructions. Extracted DNA was analyzed through viability qPCR (v-qPCR) for quantification of live bacteria with species-specific primers according to Van Holm et al (Van Holm et al., 2023). The qPCR assay was performed using a CFX96 Real-Time System (Bio-Rad, Hercules, CA, USA) and its associated software.

**Table 2:** Oral bacteria used in the biofilm model.

Species	Class
*Aggregatibacter actinomycetemcomitans* ATCC 43718 *Prevotella intermedia* ATCC 25611 *Porphyromonas gingivalis* ATCC 33277 *Fusobacterium nucleatum* ATCC 20482 *Actinomyces viscosus* DSM 43327 *Actinomyces naeslundii* ATCC 51655 *Veillonella parvula* DSM 2008 *Streptococcus mutans* ATCC 20523 *Streptococcus sobrinus* ATCC 20742 *Streptococcus oralis* DSM 20627 *Streptococcus sanguinis* LM14657 *Streptococcus gordonii* ATCC 49818 *Streptococcus mitis* DSM 12643 *Streptococcus salivarius* TOVE-R	Periodontal pathobiont Anaerobic commensal Cariogenic streptococci Commensal streptococci

### 2.7 Statistical analysis

Data from flow cytometry and v-qPCR were used to calculate probiotic viability, total viable bacterial load, biofilm ecology, and the relative abundance of each of the 14 biofilm species and probiotic strains. Statistical analyses were performed using R (version 4.2.2). Statistical tests for each experiment were selected based on the experimental design, normality of residuals, and equality of variances. Normality of residuals was assessed using the Shapiro–Wilk test, and homogeneity of variances was assessed using Levene’s test. For independent-group comparisons with normally distributed residuals, data were analyzed using one-way ANOVA followed by Tukey’s HSD *post hoc* test, or Welch’s ANOVA followed by the Games–Howell *post hoc* test when variances were unequal. When residuals deviated from normality, data were analyzed using the Kruskal–Wallis test followed by pairwise Wilcoxon rank-sum tests. When normality of residuals was violated, paired or repeated-measures data were analyzed using the Friedman test followed by pairwise Wilcoxon signed-rank tests. P-values were adjusted for multiple comparisons where appropriate, and statistical significance was set at p < 0.05.

## 3 Results

### 3.1 *In vitro* survival of probiotics exposed to toothpastes

**Figure 1:**
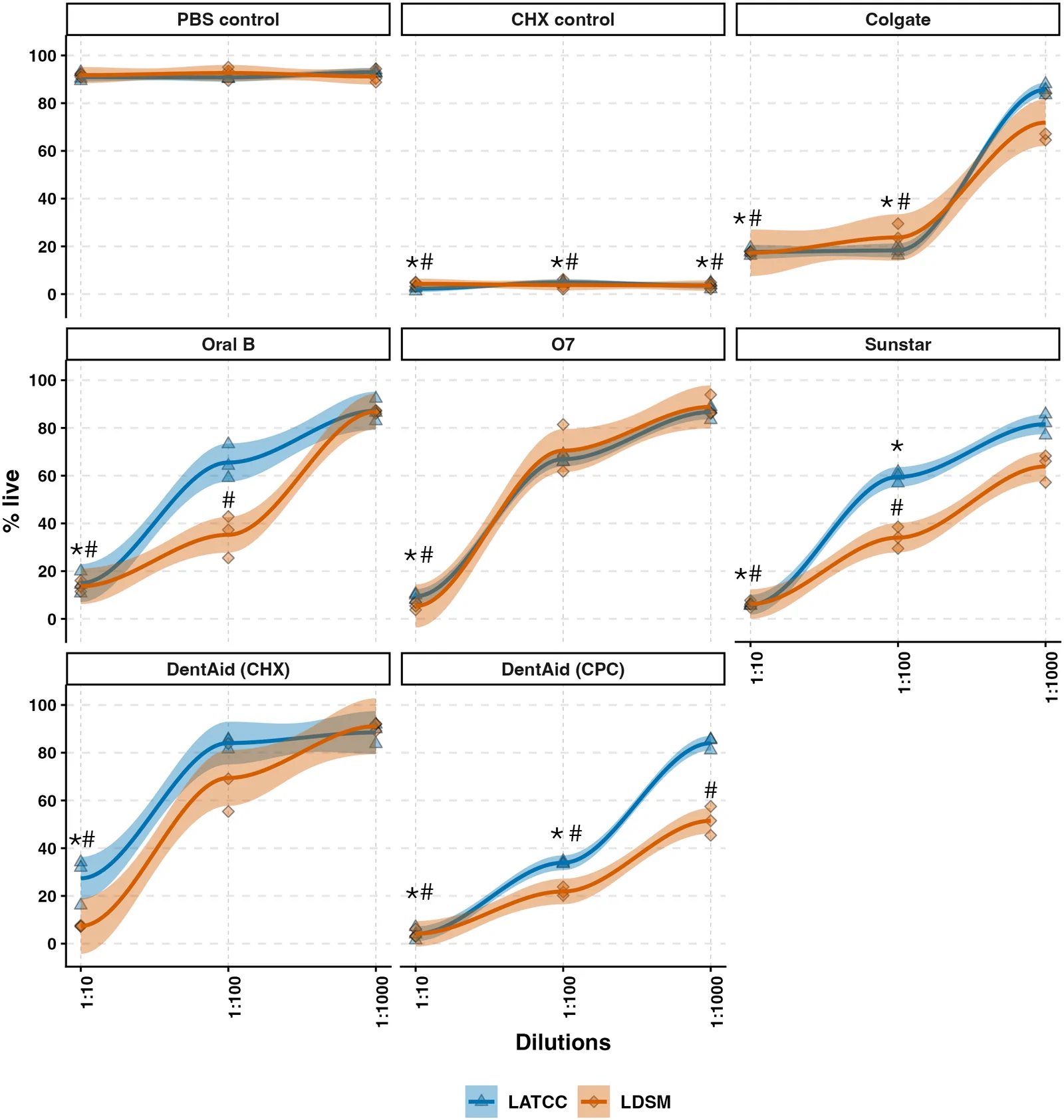
Inhibition of selected toothpastes on probiotics viability. Starting from a tenfold dilution of each toothpaste, two additional tenfold dilutions were prepared. Probiotics (*L. reuteri* ATCC and DSM; 10^8^ cells/mL) were incubated in these dilutions for 24 h. Data are expressed as the percentage of live/intact cells within the probiotic gate from flow cytometric quantification with viability stains. Results are based on three independent replicates (n = 3). Statistical analysis was performed using the Kruskal–Wallis test followed by Dunn’s test for *post hoc* comparisons with Holm correction (p < 0.05). The * symbol represents significantly lower survival of *L. reuteri* ATCC compared with the PBS control, while the # symbol represents significantly lower survival of *L. reuteri* DSM compared with the PBS control. .

To evaluate the tolerance of the probiotic strains to different toothpastes (**Table 1**), serial dilutions of each toothpaste slurry were incubated with the probiotics (**Figure 1**). At 1 in 10 (10%) dilution, all 6 toothpastes significantly reduced probiotic viability. In contrast, at a 1 in 1000 (0.1%) dilution, most toothpastes exhibited no antimicrobial effect, except for DentAid(CPC)^®^, which showed a mild killing effect on *L. reuteri* DSM. At the 1 in 100 (1%) dilution, the effects varied by product; O7^®^ and DentAid(CHX)^®^ displayed minimal killing effects on both *L. reuteri* strains, whereas OralB^®^ exerted a stronger inhibitory effect on *L. reuteri* DSM than on *L. reuteri* ATCC. Colgate^®^, Sunstar^®^, and DentAid (CPC)^®^ demonstrated pronounced antimicrobial activity against both probiotic strains, indicating clear product-specific differences.

### 3.2 *Ex vivo* survival of probiotics in saliva after brushing with toothpastes

**Figure 2:**
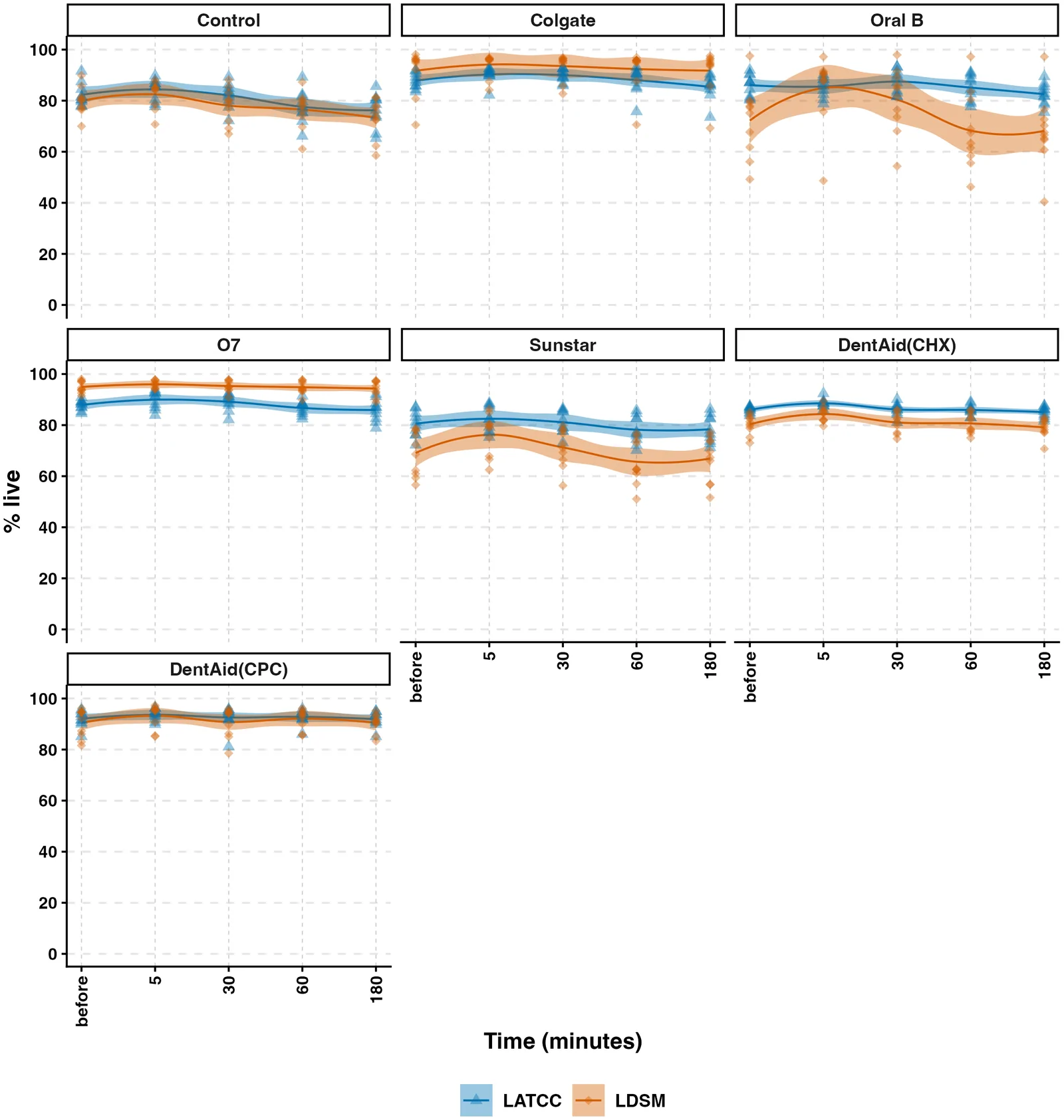
*Ex vivo* survival of probiotics in saliva after brushing with selected toothpastes. Survival of *L. reuteri* ATCC and DSM (10^8^ cells/mL) was assessed after 2 h of incubation in saliva collected from 10 volunteers who brushed with different toothpastes (brushing without any toothpaste as the control). Saliva samples were collected at baseline and at 5, 30, 60, and 180 minutes post-brushing. Data are expressed as the percentage of live probiotics relative to the initial source culture, quantified by flow cytometry with viability stains. Results are presented as mean values from ten independent donors (n = 10, Individual data points are shown as either triangles or diamonds.). Statistical differences compared with the control were determined using Wilcoxon signed-rank tests with Holm correction (p < 0.05, effect sizes, confidence intervals and exact p-values in **Supplementary Table 2**). No statistically significant differences were observed.

To evaluate the viability of the probiotics in saliva following tooth brushing with the different toothpastes, the proportion of live and intact probiotic cells was quantified using flow cytometry with a viability stain (**Figure 2**). PBS served as the negative control and consistently maintained high probiotic survival, indicating negligible antimicrobial activity. Similarly, no significant differences were detected across any of the toothpaste groups at any of the assessed time points. Both *L. reuteri* strains remained viable in saliva after brushing with each of the six different toothpastes.

### 3.3 *In vitro* adhesion to hydroxyapatite

**Figure 3:**
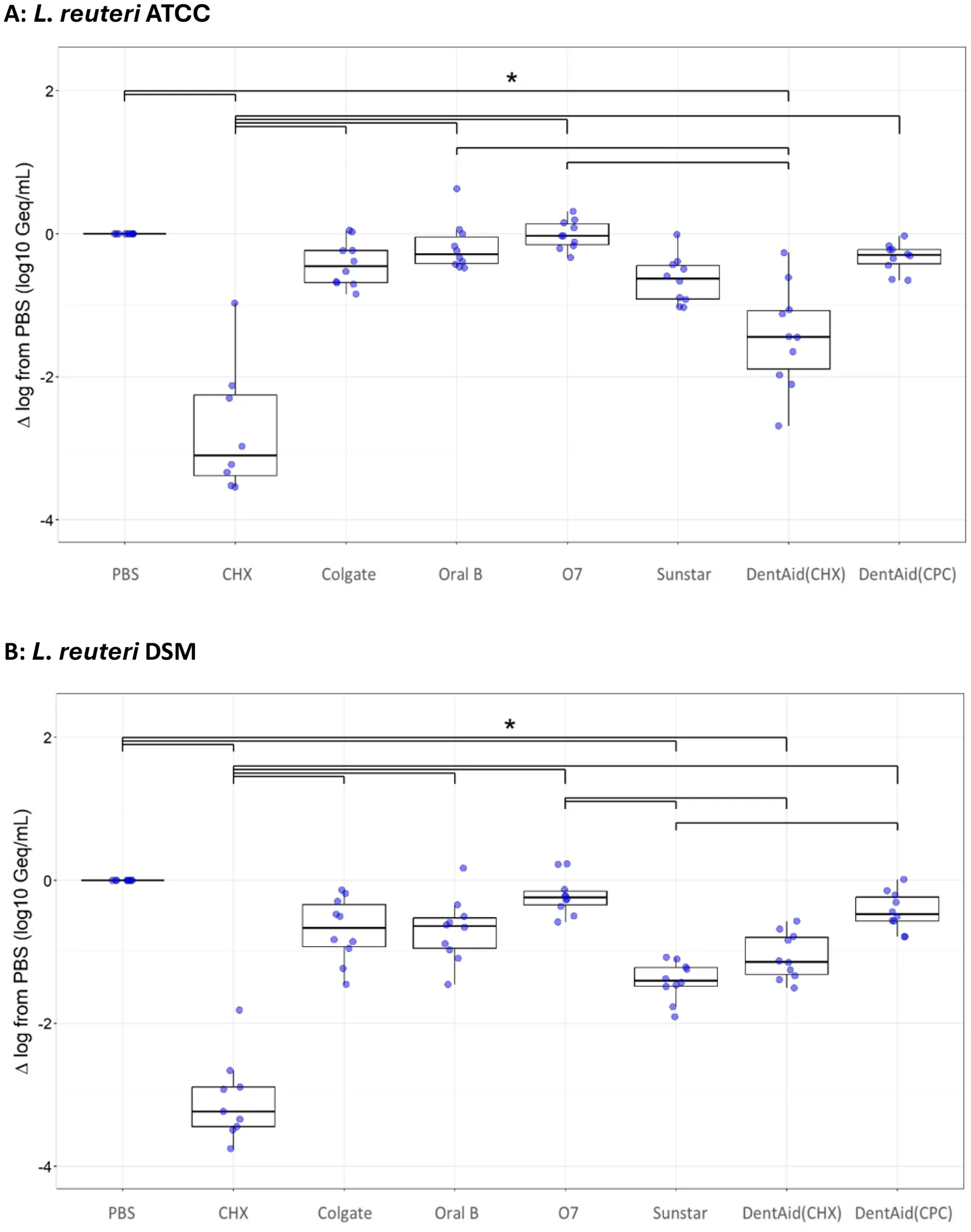
Log difference in colonization of *L. reuteri* ATCC **(A)** and *L. reuteri* DSM **(B)** to toothpaste slurry rinsed saliva coated hydroxyapatite discs when compared to the PBS-control in saliva. Purple dots show each individual donor. The * represents significant differences between conditions (p < 0.05; ANOVA followed by Tukey HSD; n = 10).

To assess the effects of the different toothpastes on *L. reuteri* probiotic adhesion, the ability of *L. reuteri* strains to adhere to saliva-coated hydroxyapatite (HA) discs was evaluated in the presence of the various toothpaste slurries (**Figures 3A, B**). The adhesion of both *L. reuteri* ATCC and DSM strains varied significantly depending on the toothpaste used. For *L. reuteri* ATCC, significant differences were observed between DentAid(CPC)^®^ and Sunstar^®^ (p < 0.001), O7 and Sunstar^®^ (p < 0.001), and O7 and DentAid(CHX)^®^ (p < 0.001). For *L. reuteri* DSM, significant differences were found between DentAid(CHX)^®^ and O7 (p < 0.001), as well as between DentAid(CHX)^®^ and Oral-B^®^ (p < 0.001). In saliva, Sunstar^®^, and DentAid(CHX)^®^ inhibited the adhesion of *L. reuteri* DSM, whereas the adhesion of *L. reuteri* ATCC was inhibited only by DentAid(CHX)^®^. Compared with the PBS control, DentAid(CHX)^®^ significantly reduced the adhesion of both *L. reuteri* ATCC and *L. reuteri* DSM (p < 0.001), while Sunstar^®^ also significantly reduced the adhesion of *L. reuteri* DSM (p < 0.001).

### 3.4 *In vitro* effects on oral biofilms

**Figure 4:**
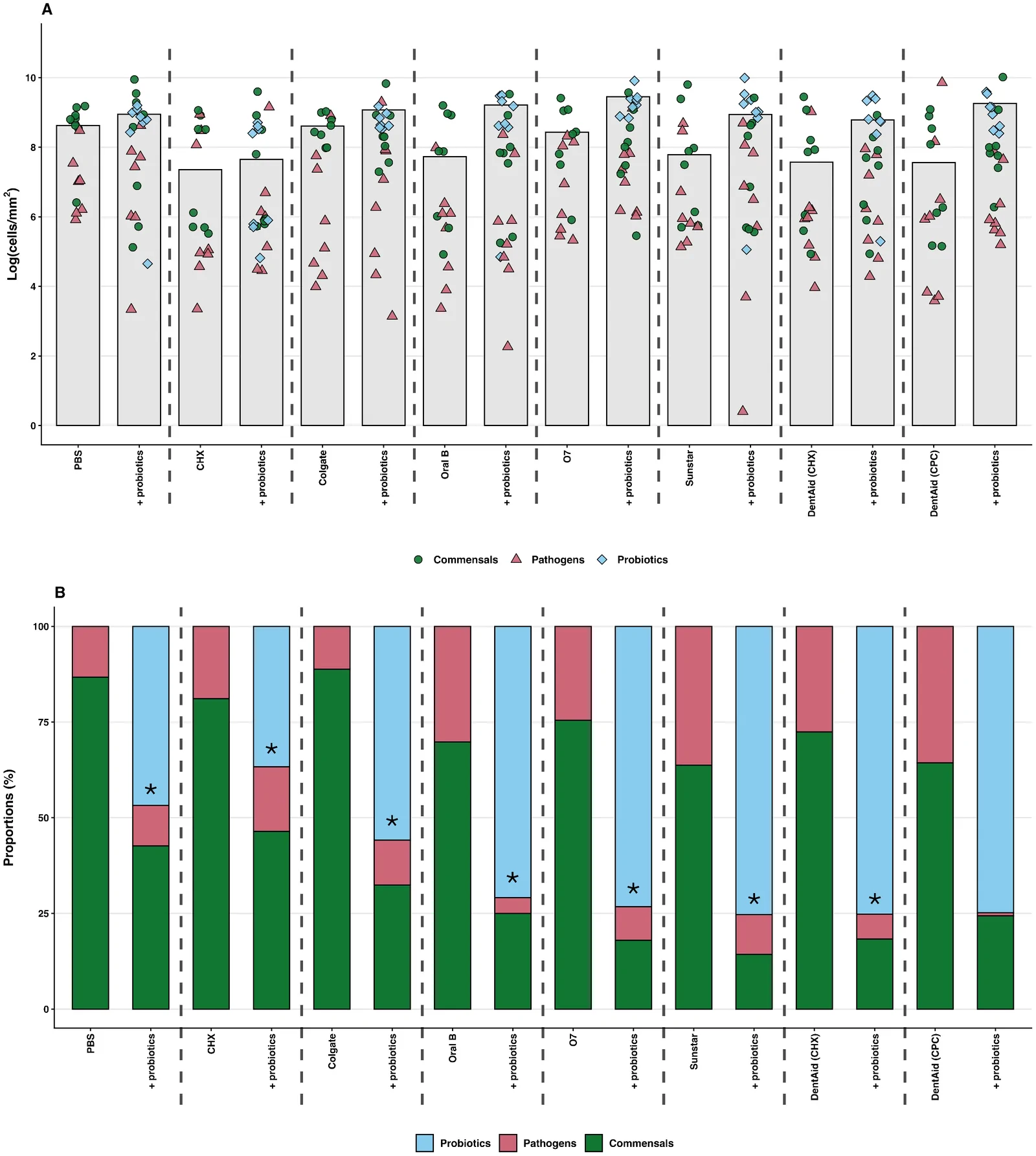
Probiotics effect on *in vitro* multispecies oral biofilms. **(A)** Total amount of live pathogens (pink), commensals (green) recovered from the biofilms, and probiotic quantity (blue; *L. reuteri* ATCC and DSM) within the biofilms. Data expressed as the base 10 logarithm of the number of cells recovered from the surface of the HAD (mm2). **(B)** Ecology of pre-treated with toothpaste biofilms plus probiotics expressed as percentages of commensals and pathobionts in total biofilm (8 replicates). The * represents significant differences between proportions of commensals and pathogens for each toothpaste in comparison with and without adding probiotics (*p* < 0.05; Friedman test followed by Wilcoxon signed-rank tests for *post hoc* comparisons; *n* = 8).

Using an *in vitro* multi-species (**Table 2**) biofilm model, the effects of brushing oral biofilms with the different toothpastes and subsequently adding *L. reuteri* strains were evaluated (**Figures 4A, B**). Brushing with toothpaste alone did not significantly affect the total biofilm load after 24 h of incubation, which may reflect recovery of the biofilm following treatment. However, after probiotic addition, the total biofilm mass increased, with approximately half of the biomass consisting of the probiotic strains themselves. The microbial composition shifted markedly only when probiotics were applied after brushing with the different toothpastes, showing a reduced proportion of pathogenic species, whereas brushing with toothpaste alone did not alter the biofilm composition.

## 4 Discussion

This study showed that toothpastes influence probiotic viability and colonization of HA surfaces negatively, but do not impair probiotic incorporation into multispecies biofilms. These observations indicate that probiotics can be used concomitantly with antiseptic-containing toothpastes. Although the antimicrobial and anti-inflammatory effects of probiotics are increasingly investigated *in vitro*, the interaction between toothpastes and probiotic efficacy has not previously been explored. This knowledge is clinically relevant because, *in vivo*, a toothpaste may act synergistically, antagonistically, or have no effect on the activity of oral probiotics. The present study, therefore, aimed to elucidate if and how different toothpastes affect the viability, adhesion, and functional efficacy of probiotics in modulating oral biofilms.

Given that the antiseptic compounds of toothpastes may impair probiotic viability, the survival of two *L. reuteri* strains was assessed in diluted toothpaste slurries. Toothpaste-, concentration-, and strain-dependent reductions in the viability of probiotics strains were observed. These data are in line with what has been shown for antiseptic mouthrinses (Lauwens et al., 2025). Although it is assumed that this effect can be attributed to the specific antiseptic in the toothpastes, it remains to be determined if other formulation-dependent factors play a role.

This is clinically relevant because probiotics for oral health are typically administered as lozenges that dissolve in saliva before reaching their target sites in the oral cavity. Residual antiseptic compounds from toothpaste may therefore eliminate probiotic bacteria before they can colonize and exert their beneficial effects. However, despite the findings from the previously mentioned *in vitro* experiments, no significant reduction in probiotic viability was observed at any time point after brushing with any of the tested toothpastes.

This might suggest that the antiseptic compounds present in toothpastes are quickly removed or neutralized in saliva, thereby minimizing potential adverse effects on probiotic survival. Although not directly assessing probiotics, an *in vivo* study by Otten et al (Otten et al., 2012). showed that the use of an anti-microbial toothpaste reduced bacterial viability significantly in plaque, but the antimicrobial activity of the toothpaste was not detectable in saliva. Our data are in contrast to those of Lauwens et al (Lauwens et al., 2025), who looked at *ex vivo* survival of probiotics in saliva after using antiseptic mouthrinses. In contrast to toothpaste, certain antiseptic mouthrinses could reduce probiotic survival in saliva up to 30 minutes after their use. This difference might be related to differences in formulation between toothpastes and mouth rinses. Furthermore, emerging evidence supports postbiotics as a potential alternative to live probiotics in oral health applications, as their effects are not dependent on bacterial viability and may therefore overcome limitations associated with antimicrobial oral care products (Heidari et al., 2025).

Since colonization of intra-oral hard surfaces might be of importance for oral probiotics to exert their effects, the influence of toothpastes on hard surface colonization was investigated. Not all toothpaste formulations affected the adhesion of *L. reuteri* strains to HA discs in the same manner. Sunstar® and DentAid(CHX)® inhibited the adhesion of *L. reuteri* ATCC, whereas the adhesion of *L. reuteri* DSM was only inhibited by DentAid(CHX)®. The other toothpaste formulations neither improved nor reduced adhesion. These toothpaste specific and probiotic-strain-specific effects are also reported for antimicrobial mouth rinses (Lauwens et al., 2025).

To achieve an optimal probiotic effect in the oral cavity, antimicrobial components in toothpastes should target oral pathobionts without negatively affecting probiotic viability within biofilms. Previous studies have shown that certain antiseptic mouthrinses can either increase or decrease the proportion of probiotics in oral biofilms (Lauwens et al., 2025). In our experiments, the total biofilm biomass increased under all conditions when probiotics were added. Compositional analysis further revealed that none of the tested formulations reduced the proportion of probiotics in the multispecies biofilm. This finding contrasts with previous reports on mouthrinses (Lauwens et al., 2025). At present, it remains unclear which specific toothpaste or mouth rinse components are responsible for the increased incorporation of probiotics into the biofilms. Previous studies have demonstrated that the effects of mouthrinses on oral biofilms are influenced by both the specific antiseptic agent and the formulation matrix in which it is incorporated (Zayed et al., 2022, Zayed et al., 2024; Lauwens et al., 2025).

Our findings complement recent clinical evidence on probiotic-containing toothpastes. A recent systematic review and meta-analysis demonstrated that lactic acid bacteria-based toothpastes can improve plaque control and gingival inflammation, with periodontal benefits becoming more apparent following prolonged use (Choi and Park, 2025). However, the reported clinical outcomes varied considerably depending on the probiotic strain, treatment protocol, and patient population (Choi and Park, 2025). The present findings suggest that the compatibility of probiotics with toothpaste formulations should also be considered when developing and evaluating probiotic oral care products, as maintaining probiotic survival and colonization potential is a prerequisite for their intended clinical effects.

It should be noted as a limitation of the study that the current study used a 14-species biofilm model, which is less complex than the natural oral microbiome. As these experiments were conducted under *in vitro* and *ex vivo* conditions, their results may not fully approach the *in vivo* oral situation. In addition, the 2-hour probiotic incubation period before biofilm re-growth represents a single exposure time point and may not fully mimic the repeated daily exposure that would occur during regular probiotic use. Repeated probiotic exposure and longer incubation periods may provide greater opportunity for probiotic incorporation into biofilms and for the development of potential beneficial effects. Future studies should therefore evaluate the impact of repeated probiotic administration over longer periods using more complex biofilm models and clinical study designs.

Another limitation is that only saliva was used as the biological sample. Although saliva provides a clinically relevant environment that closely resembles the natural oral habitat, containing host-derived proteins, nutrients, and microorganisms involved in biofilm formation and bacterial colonization (Darrene and Cecile, 2016), it cannot fully replicate the complexity and dynamics of the *in vivo* oral environment. Nevertheless, saliva is a non-invasive and reproducible sample that reflects the oral microbial community, making it well suited for *ex vivo* oral microbiological studies. Future studies should validate these findings using more complex *in vitro* models.

Another consideration is that individual salivary characteristics were not specifically evaluated. Saliva exhibits considerable variation in its protein and antimicrobial peptide profiles between individuals and over time, and factors such as salivary flow, pH, buffering capacity, and the concentrations of antimicrobial proteins and peptides may influence probiotic survival and colonization potential (Grant et al., 2019; Gallo et al., 2024).

Future clinical studies should therefore assess the colonization potential and compatibility of oral probiotics following brushing with different toothpaste formulations to validate these findings under real-world conditions. Moreover, future randomized clinical trials should investigate whether different oral hygiene procedures influence the clinical efficacy of oral probiotics and determine the optimal way to integrate probiotics into daily oral hygiene routines. Such studies are essential for developing evidence-based recommendations regarding the timing of probiotic administration and the selection of compatible oral hygiene products to maximize their clinical benefits.

## 5 Conclusion

In conclusion, the findings of this study suggest that, unlike antiseptic mouthrinses, the tested toothpastes do not negatively affect the survival and colonization of the probiotics in modulating biofilms, although certain formulations reduced probiotic viability and limited their adhesion potential *in vitro*. The *ex vivo* results further demonstrated that toothbrushing with these toothpastes did not impair probiotic survival in saliva, supporting the compatibility and safe use of oral probiotics following routine brushing with toothpaste.

## Data Availability

The raw data supporting the conclusions of this article will be made available by the authors, without undue reservation.
