# Supplementary material for: Compatibility of toothpastes with probiotics for periodontal health: an in vitro and ex vivo study
Source: Front Cell Infect Microbiol. 2026 Sep 11;16:1829275. doi: 10.3389/fcimb.2026.1829275 (PMC13612308; doi:10.3389/fcimb.2026.1829275)
Supplement: Supplementary file 1 [file DataSheet1.pdf]

Supplemental Table1: Used primers and probes

Species		Primers & Probe (Final concentrations)	Amplicon length (bp)	Target
Aggregatibacter actinomycetemcomitans	Forward	CGG TGT CGA TTT GGG GAT TGG(300 nM)	237	16S rRNA gene
	Reverse	TGC AGC ACC TGT CTC AAA GC (300 nM)		
	Probe	AGA ACT CAG AGA TGG GTT TGT GCC TTA GGG (100 nM)		
Prevotella intermedia	Forward	TGT GCC CYT TTG CAT TTA CCC TTC(300 nM)	216	16S rRNA gene
	Reverse	CAC CAT GAA TTC CGC ATA CG (900 nM)		
	Probe	TGG CGG ACT TGA GTG CAC GC (200 nM)		
Porphyromonas gingivalis	Forward	CCG TAA GAA TAA GCA TCG GCT AAC TC(300 nM)	195	16S rRNA gene
	Reverse	CAC GAA TTC CGC CTG C (300 nM)		
	Probe	CAC TGA ACT CAA GCC CGG CAG TTT CAA (100 nM)		
Fusobacterium nucleatum	Forward	GGA TTT ATT GGG CGT AAA GC (300 nM)	191	16S rRNA gene
	Reverse	ATC TGT CCA GTA AGC TGG CTT CC (300 nM)		
	Probe	CTC TAC ACT TGT AGT TCC G (300 nM)		
Streptococcus mutans	Forward	GCC TAC AGC TCA GAG ATG CTA TTC T (900 nM)	114	gtfB gene
	Reverse	GCC ATA CAC CAC TCA TGA ATT GA (900 nM)		
	Probe	TGG AAA TGA CGG TCG CCG TTA TGA A (100 nM)		
Streptococcus sobrinus	Forward	AAA TAC GGC CAG TGC CAA AG (200 nM)	165	gtfT gene
	Reverse	CCA GCC TGA GAT TCA GCT TGT (200 nM)		
	Probe	CCT GCT CCA GCG ACA AAG GCA GC (250 nM)		
Actinomyces naeslundii	Forward	TCG AAA CTC AGC AAG TAG CCG (200 nM)	96	gene encoding an unknown protein
	Reverse	AGA GGA GGG CCA CAA AAG AAA (200 nM)		
	Probe	GGG TAC TCT AGT CCA AAC TGG CGG ATA GCG (100 nM)		
Actinomyces viscosus	Forward	GTG AAG GAG CCA GCT TGC TGG TTC TG (200 nM)	155	16S rRNA gene
	Reverse	CGG AAC AAA CCT TTC CCA GGC (200 nM)		
	Probe	ATG AGT GGC GAA CGG GTG AGT AAC (125 nM)		
Veillonella parvula	Forward	GAC GAA AGT CTG ACG GAG CA (200 nM)	171	16S rRNA gene
	Reverse	TGC CAC CTA CGT ATT ACC GC (200 nM)		
	Probe	AGC TCT GTT AAT CGG GAC GAA AGG C (125 nM)		
Streptococcus oralis	Forward	ACC AGC AGA TAC GAA AGA AGC AT (400 nM)	229	*gtfR* gene
	Reverse	AGG TTC GGG CAA GCG ATC TTT CT (400 nM)		
	Probe	AAG GCT GCT GTT GCT GAA GAA GT (100 nM)		
Streptococcus sanguinis	Forward	CAA AAT TGT TGC AAA TCC AAA GG (600 nM)	75	*gtfP* gene
	Reverse	GCT ATC GCT CCC TGT CTT TGA (600 nM)		
	Probe	AAA GAA AGA TCG CTT GCC AGA ACC GG (100 nM)		
Streptococcus gordonii	Forward	GAA GAA CTG GGT AGC GAT TGC T (400 nM)	262	*gtfG* gene
	Reverse	GTT AGC TGT TGG ATT GGT TGC C (400 nM)		
	Probe	AGA ACA GTC CGC TGT TCA GAG CAA (100 nM)		
Streptococcus mitis	Forward	GGC TCG TAG TCT GGA GAT GG (600 nM)	133	16S rRNA gene
	Reverse	TAG GTC GTC GTC CCA AGG AA (600 nM)		
	Probe	CGA AGA GCA CCA ATA GCA CCT CCC (140 nM)		
Streptococcus salivarius	Forward	GAC GAT GAC TGT CAA CTT GAC AC (400 nM)	247	*Dextranase* gene
	Reverse	ACC GTA ACG TGG GAA AAC TG (400 nM)		
	Probe	GTA GCG TCA GAG TGG TTG AC (100 nM)		
L. reuteri DSM17938	Forward	TTAAGGATGCAAACCCGAAC (300 nM)	177	gene encoding an unknown protein
	Reverse	CCTTGTCACCTGGAACCACT (300 nM)		
	Probe	FAM- ATGACCTTGTAGGAGCCGTCTGGATAC -Tamra (100 nM)		
L. reuteri ATCC PTA5289	Forward	GACAGTGGCTAAACGCCTTC (300 nM	200	gene encoding an unknown protein
	Reverse	AATTCCACTTGCCATCTTCG (300 nM)		
	Probe	FAM-TTGGTAGGCTTGTTGAAAATCGCTA-TARMA (100 nM)		

Supplementary table 2: Effect sizes, confidence intervals and p-values of figure 2

toothpaste	probiotic	comparison	n	mean_before	mean_comparison	median_before	median_comparison	mean_diff	median_diff	cohens_d	wilcox_statistic	p_value	conf_low	conf_high	estimate	p_adjusted_global
Oral B	LATCC	5 vs before	10	0.860876574	0.855188833	0.870994577	0.864816068	-0.005687741	-0.015961231	-0.240103214	35	0.47553266	-0.012450065	0.023728702	0.007709317	1
Oral B	LATCC	30 vs before	10	0.860876574	0.874689693	0.870994577	0.881666845	0.013813119	0.015642802	0.507876086	14	0.185143724	-0.033846363	0.008280509	-0.016125789	1
Oral B	LATCC	60 vs before	10	0.860876574	0.851098197	0.870994577	0.860588094	-0.009778377	-0.002076633	-0.276369219	33	0.610298656	-0.013108669	0.042929667	0.003339762	1
Oral B	LATCC	180 vs before	10	0.860876574	0.825405696	0.870994577	0.82382313	-0.035470878	-0.023481391	-1.036907281	55	0.005921537	0.010264703	0.062068203	0.029214649	0.331606073
Oral B	LDSM	5 vs before	10	0.722103713	0.84953038	0.762794919	0.896971102	0.127426667	0.107306611	0.65213494	9	0.066545721	-0.260203577	0.013607661	-0.124130874	1
Oral B	LDSM	30 vs before	10	0.722103713	0.803834177	0.762794919	0.833727105	0.081730465	0.057884269	0.465911757	10	0.083131143	-0.211446787	0.044331451	-0.061250982	1
Oral B	LDSM	60 vs before	10	0.722103713	0.682790396	0.762794919	0.654157033	-0.039313316	-0.038039454	-0.296800774	40	0.221271816	-0.069257968	0.130653736	0.050598962	1
Oral B	LDSM	180 vs before	10	0.722103713	0.682053142	0.762794919	0.670944286	-0.040050571	-0.07875699	-0.36519866	41	0.185143724	-0.052850812	0.113406709	0.065114231	1
DentAid(CPC)	LATCC	5 vs before	10	0.920667125	0.936205663	0.922930871	0.940419348	0.015538538	0.013176264	0.740476334	11	0.102917539	-0.033779311	0.003163691	-0.014069581	1
DentAid(CPC)	LATCC	30 vs before	10	0.920667125	0.925643528	0.922930871	0.940960042	0.004976403	0.006985439	0.225082548	18	0.358951436	-0.021197288	0.012880427	-0.006620813	1
DentAid(CPC)	LATCC	60 vs before	10	0.920667125	0.928315734	0.922930871	0.933345216	0.007648608	0.007822005	0.585186824	12	0.126278929	-0.019315293	0.002845922	-0.007424571	1
DentAid(CPC)	LATCC	180 vs before	10	0.920667125	0.918783685	0.922930871	0.923642959	-0.00188344	-0.000731203	-0.092804284	31	0.759765826	-0.015266286	0.017076474	0.000859114	1
DentAid(CPC)	LDSM	5 vs before	10	0.90478618	0.932562033	0.933917353	0.954902659	0.027775853	0.016205568	0.924849371	2	0.010826921	-0.051052164	-0.006986291	-0.022645287	0.552172985
DentAid(CPC)	LDSM	30 vs before	10	0.90478618	0.907928815	0.933917353	0.941386366	0.003142635	0.003523498	0.045329964	19	0.414823067	-0.039435934	0.069169891	-0.015925072	1
DentAid(CPC)	LDSM	60 vs before	10	0.90478618	0.921307638	0.933917353	0.935127993	0.016521459	4.27417E-06	0.564616572	19	0.414823067	-0.040923059	0.005759646	-0.015914103	1
DentAid(CPC)	LDSM	180 vs before	10	0.90478618	0.904815559	0.933917353	0.910641635	2.93798E-05	-0.001783807	0.000796844	29	0.918812683	-0.027446455	0.03040772	0.003452696	1
O7	LATCC	5 vs before	10	0.878652302	0.899601426	0.882782854	0.91207508	0.020949124	0.025392754	0.826908411	7	0.041491087	-0.041079198	-0.001219212	-0.02330504	1
O7	LATCC	30 vs before	10	0.878652302	0.891511545	0.882782854	0.902130227	0.012859243	0.0080255	0.449789864	9	0.066545721	-0.034383874	0.008919861	-0.011100296	1
O7	LATCC	60 vs before	10	0.878652302	0.867079499	0.882782854	0.874783333	-0.011572803	-0.008716782	-0.548018207	42	0.153576397	-0.004822659	0.027441902	0.010400876	1
O7	LATCC	180 vs before	10	0.878652302	0.859200813	0.882782854	0.864821947	-0.019451489	-0.023848394	-0.604525464	43	0.126278929	-0.003448295	0.045933986	0.020170311	1
O7	LDSM	5 vs before	10	0.949455665	0.959848332	0.94322352	0.960109057	0.010392667	0.00937825	0.994827754	4	0.019058892	-0.019219108	-0.002043524	-0.010233237	0.933885688
O7	LDSM	30 vs before	10	0.949455665	0.95313823	0.94322352	0.949987548	0.003682565	0.004360241	0.338834411	18	0.358951436	-0.012350811	0.004753377	-0.003951882	1
O7	LDSM	60 vs before	10	0.949455665	0.948324275	0.94322352	0.944098058	-0.00113139	-0.001482354	-0.129348703	30	0.838463782	-0.005582313	0.008438176	0.001475976	1
O7	LDSM	180 vs before	10	0.949455665	0.94331693	0.94322352	0.947881012	-0.006138736	9.4278E-05	-0.354686037	33	0.610298656	-0.006288363	0.021120908	0.003478213	1
Colgate	LATCC	5 vs before	10	0.878198189	0.902698299	0.876843152	0.910144214	0.02450011	0.023774391	0.850925507	6	0.032312911	-0.046977596	-0.002586358	-0.023718986	1
Colgate	LATCC	30 vs before	10	0.878198189	0.900078783	0.876843152	0.901896245	0.021880594	0.022924739	0.668140589	10	0.083131143	-0.046447331	0.004080119	-0.02197737	1
Colgate	LATCC	60 vs before	10	0.878198189	0.880389064	0.876843152	0.899773874	0.002190875	-0.000221744	0.051591844	25	0.838463782	-0.03596544	0.026372681	-0.003368687	1
Colgate	LATCC	180 vs before	10	0.878198189	0.854138367	0.876843152	0.862405311	-0.024059822	-0.021512533	-0.618177194	45	0.083131143	-0.004245033	0.05733654	0.022290494	1
Colgate	LDSM	5 vs before	10	0.916902811	0.941526889	0.95559305	0.961007764	0.024624078	0.006569106	0.530206558	15	0.221271816	-0.067011283	0.004240782	-0.014034759	1
Colgate	LDSM	30 vs before	10	0.916902811	0.935342119	0.95559305	0.957236429	0.018439308	0.004550195	0.42776482	17	0.30806323	-0.056611737	0.008446362	-0.008926471	1
Colgate	LDSM	60 vs before	10	0.916902811	0.923818622	0.95559305	0.956099928	0.006915811	-0.001727585	0.252777976	27	1	-0.031726476	0.012231019	-0.000292634	1
Colgate	LDSM	180 vs before	10	0.916902811	0.91752602	0.95559305	0.945318942	0.000623209	-0.009358258	0.029212365	33	0.610298656	-0.018078365	0.01349932	0.00442051	1
Control	LATCC	5 vs before	10	0.822681565	0.844227889	0.808930507	0.856124343	0.021546324	0.022372314	0.670573091	10	0.083131143	-0.046433693	0.002527364	-0.022172848	1
Control	LATCC	30 vs before	10	0.822681565	0.820740676	0.808930507	0.827245657	-0.001940889	-0.009473332	-0.044391296	27	1	-0.032203563	0.029840212	-0.000723003	1
Control	LATCC	60 vs before	10	0.822681565	0.77608167	0.808930507	0.772490169	-0.046599895	-0.044657149	-0.986059244	49	0.032312911	0.007044568	0.08041013	0.045396631	1
Control	LATCC	180 vs before	10	0.822681565	0.762289877	0.808930507	0.774640217	-0.060391688	-0.068283943	-1.048833287	49	0.032312911	0.016948934	0.104800798	0.065183681	1
Control	LDSM	5 vs before	10	0.799126768	0.82469983	0.801214299	0.846058796	0.025573062	0.029883775	0.798197987	9	0.066545721	-0.051069181	0.001802419	-0.025456866	1
Control	LDSM	30 vs before	10	0.799126768	0.780859279	0.801214299	0.793945911	-0.018267489	-0.014164518	-0.414356287	38	0.30806323	-0.014310156	0.053032475	0.014085563	1
Control	LDSM	60 vs before	10	0.799126768	0.765171907	0.801214299	0.780994147	-0.033954861	-0.037277474	-0.657325912	45	0.083131143	-0.008584533	0.071951254	0.037213262	1
Control	LDSM	180 vs before	10	0.799126768	0.733248281	0.801214299	0.747092759	-0.065878487	-0.059686325	-1.2876642	52	0.014432824	0.023607316	0.103578531	0.061822348	0.721641197
DentAid(CHX)	LATCC	5 vs before	10	0.859899492	0.885430908	0.862058196	0.885160271	0.025531416	0.023848132	1.077286965	0	0.005921537	-0.042114931	-0.008731666	-0.021910352	0.331606073
DentAid(CHX)	LATCC	30 vs before	10	0.859899492	0.861362108	0.862058196	0.863068642	0.001462616	0.003291948	0.046564983	27	1	-0.026676135	0.021002207	-0.000566834	1
DentAid(CHX)	LATCC	60 vs before	10	0.859899492	0.859328892	0.862058196	0.859604897	-0.0005706	-0.008823628	-0.022360307	33	0.610298656	-0.019017942	0.016299431	0.004085994	1
DentAid(CHX)	LATCC	180 vs before	10	0.859899492	0.851004684	0.862058196	0.856700799	-0.008894807	-0.010556693	-0.374525552	40	0.221271816	-0.011559806	0.025822927	0.009825239	1
DentAid(CHX)	LDSM	5 vs before	10	0.802897077	0.84331922	0.808240015	0.848459528	0.040422143	0.031359946	1.408926035	0	0.005921537	-0.063605285	-0.018070919	-0.04137215	0.331606073
DentAid(CHX)	LDSM	30 vs before	10	0.802897077	0.810423547	0.808240015	0.817211885	0.00752647	0.013816066	0.177003101	22	0.610298656	-0.042285956	0.028326386	-0.008878038	1
DentAid(CHX)	LDSM	60 vs before	10	0.802897077	0.806492458	0.808240015	0.802968869	0.003595381	-0.00270108	0.102221246	29	0.918812683	-0.030099571	0.022760074	0.001066895	1
DentAid(CHX)	LDSM	180 vs before	10	0.802897077	0.791025279	0.808240015	0.796751821	-0.011871798	-0.008277305	-0.383929115	38	0.30806323	-0.011942887	0.035028597	0.01171628	1
Sunstar	LATCC	5 vs before	10	0.805549227	0.824835646	0.806444306	0.823253168	0.019286419	0.008033962	0.545511709	13	0.153576397	-0.049716658	0.00363608	-0.00947756	1
Sunstar	LATCC	30 vs before	10	0.805549227	0.811171288	0.806444306	0.8042825	0.005622061	-0.000296665	0.187733068	24	0.759765826	-0.029091137	0.02039732	-0.007727397	1
Sunstar	LATCC	60 vs before	10	0.805549227	0.782084457	0.806444306	0.769238582	-0.02346477	-0.022570327	-1.180140248	55	0.005921537	0.00663007	0.040462371	0.022965325	0.331606073
Sunstar	LATCC	180 vs before	10	0.805549227	0.783563035	0.806444306	0.772650361	-0.021986192	-0.023595002	-0.818634697	48	0.041491087	0.000351286	0.045639034	0.022388907	1
Sunstar	LDSM	5 vs before	10	0.690794276	0.762212756	0.704742802	0.791050386	0.07141848	0.061039581	1.111084631	0	0.005921537	-0.133921502	-0.031468989	-0.061034195	0.331606073
Sunstar	LDSM	30 vs before	10	0.690794276	0.712594854	0.704742802	0.717863213	0.021800578	0.017173921	0.476419314	12	0.126278929	-0.062878615	0.014532328	-0.020422142	1
Sunstar	LDSM	60 vs before	10	0.690794276	0.656547066	0.704742802	0.627101409	-0.03424721	-0.030368863	-0.938428693	49	0.032312911	0.0047962	0.063655853	0.030917782	1
Sunstar	LDSM	180 vs before	10	0.690794276	0.668993236	0.704742802	0.685748145	-0.02180104	-0.026187609	-0.735160586	45	0.083131143	-0.004979454	0.0410769	0.026195161	1
